# Absorption Profile of (Poly)Phenolic Compounds after Consumption of Three Food Supplements Containing 36 Different Fruits, Vegetables, and Berries

**DOI:** 10.3390/nu9030194

**Published:** 2017-02-26

**Authors:** Letizia Bresciani, Daniela Martini, Pedro Mena, Michele Tassotti, Luca Calani, Giacomo Brigati, Furio Brighenti, Sandra Holasek, Daniela-Eugenia Malliga, Manfred Lamprecht, Daniele Del Rio

**Affiliations:** 1 The Laboratory of Phytochemicals in Physiology, Human Nutrition Unit, Department of Food & Drug, University of Parma, 43125 Parma, Italy; letizia.bresciani@unipr.it (L.B.); daniela.martini@unipr.it (D.M.); pedromiguel.menaparreno@unipr.it (P.M.); michele.tassotti@studenti.unipr.it (M.T.); giacomo.brigati@studenti.unipr.it (G.B.); furio.brighenti@unipr.it (F.B.); 2 Department of Food & Drug, University of Parma, 43124 Parma, Italy; luca.calani@unipr.it; 3 Institute of Pathophysiology and Immunology, Medical University of Graz, A-8010 Graz, Austria; sandra.holasek@medunigraz.at; 4 Division of Cardiac Surgery, Department of Surgery, Medical University of Graz, A-8010 Graz, Austria; daniela-eugenia.martin@medunigraz.at; 5 Institute of Physiological Chemistry, Medical University of Graz, A-8010 Graz, Austria; manfred.lamprecht@medunigraz.at; 6 Green Beat—Institute of Nutrient Research and Sport Nutrition, 8042 Graz, Austria; 7 The Need for Nutrition Education/Innovation Programme (NNEdPro), Global Centre for Nutrition and Health, St John’s Innovation Centre, Cambridge CB4 0WS, UK

**Keywords:** fruit and vegetables, capsules, (poly)phenolic compounds, absorption

## Abstract

The market of plant-based nutraceuticals and food supplements is continuously growing due to the increased consumer demand. The introduction of new products with relevant nutritional characteristics represents a new way of providing bioactive compounds and (poly)phenols to consumers, becoming a strategy to ideally guarantee the health benefits attributed to plant foodstuffs and allowing the increase of daily bioactive compound intake. A paramount step in the study of nutraceuticals is the evaluation of the bioavailability and metabolism of their putatively active components. Therefore, the aim of the present study was to investigate the absorption profile of the (poly)phenolic compounds contained in three different plant-based food supplements, made of 36 different plant matrices, which were consumed by 20 subjects in an open one-arm study design. Blood samples were collected at baseline and 1, 2, 5, and 10 h after capsule intake. Twenty quantifiable metabolites deriving from different (poly)phenolic compounds were identified. Results showed that the consumption of the three capsules allowed the effective absorption of several (poly)phenolic compounds and metabolites appearing at different times in plasma, thereby indicating different absorption profiles. The capsules thus ensured potential health-promoting molecules to be potentially available to target tissues and organs.

## 1. Introduction

Daily consumption of five portions or at least 400 g of fruits and vegetables is promoted in many national and international dietary guidelines and by public policies [1,2]. An adequate consumption of fruit and vegetables has been associated with human health promotion thanks to their role in chronic disease prevention [3]. Conversely, it has been estimated that up to 2635 million deaths per year can be attributable to low fruit and vegetable consumption [4]. In detail, several scientific studies report the correlation between fruit and vegetable consumption with a reduction in all-cause mortality [5], a decrease in overall cancer risk [6], and prevention of metabolic diseases like type II diabetes [7,8]. Many national nutrition surveys have shown that the minimum population goal of 400 g of fruit and vegetables per day established by FAO/WHO is not easily met, especially in selected groups of population such as teenagers [9,10]. However, nowadays, consumers are more aware of the health benefits associated with a healthy lifestyle, including high consumption of fruit and vegetables, increasing their will to buy products rich in bioactive compounds [11]. In this context, consumer health demands have boosted the market of plant-based nutraceuticals and food supplements, encouraging the introduction of new products with relevant nutritional characteristics. These products represent new ways of providing bioactive compounds to consumers, becoming a relevant strategy to ideally guarantee the health benefits attributed to plant foodstuffs [12] and allowing the increase of daily bioactive compound intake.

The nutritional relevance of fruit and vegetables on human health could be attributed to their dietary fiber content, mainly soluble, as well as to a large range of micronutrients, including carotenoids, vitamins (mainly vitamin C, and folate), and minerals (as potassium, calcium, and magnesium). In addition, fruit and vegetables are well-recognized sources of non-nutrient bioactive compounds, also called “phytochemicals”, among which (poly)phenolic compounds are the predominant and the most investigated [13,14,15,16,17]. The term (poly)phenol includes a number of different chemical structures, including flavonoids and related compounds, but also refers to hydroxycinnamates and phenolic acids, which have only one phenolic ring [18].

A growing amount of experimental research is trying hard to demonstrate the beneficial role of (poly)phenols in humans, trying to unravel the biological mechanisms behind the many proposed effects. Daily consumption of (poly)phenols has been related to reduction of inflammation, hypertension, risk of cardiovascular, metabolic, neurodegenerative diseases, and cancer [18,19,20,21,22]. An essential part of the scientific research in vivo is represented by the investigation of the absorption, metabolism and bioavailability of (poly)phenolic compounds after human intake. In fact, the large structure variety of this class of phytochemicals, together with the food matrices, complex human metabolic pathways and the role of human intestinal microbiota, can deeply influence the metabolism of (poly)phenol compounds, and as a consequence, the availability of their potentially beneficial metabolites to the human body’s internal compartments.

The aim of the present study was to investigate the absorption profile of the (poly)phenolic compounds contained in three different plant-based food supplements, made of 36 different vegetal matrices, recently characterized for their (poly)phenol content [23], designed to integrate and increase the daily intake of dietary phenolics. The supplements have been previously shown to exert wide biological effects in diverse health conditions [24,25,26,27,28].

## 2. Materials and Methods

### 2.1. Subjects

The sample size of our study was based on the number of volunteers usually recruited for bioavailability studies involving (poly)phenolic compounds. However, seven subjects would have provided sufficient power (α error of 0.05, 80% power) to detect a minimum increase of 40 nmol/L in 2 h in the absorption kinetics of flavan-3-ols, based on available published data [29]. Twenty participants were recruited from the Graz region (comprising a radius of 50 km around the center of Graz, Austria) to take part in the study. Selected participants were 9 males (m) and 11 females (f) with the following characteristics: age 34.3 ± 9.9 (m) and 32.9 ± 9.1 (f), weight 71.9 ± 11.7 (m) and 63.6 ± 9.4 (f) kg, height 174 ± 9 (m) and 167 ± 9 (f) cm and BMI 23.2 ± 3.0 (m) and 22.8 ± 3.05 (f) (kg/m^2^) (data expressed as mean ± SD). The volunteers had to meet all the inclusion and exclusion criteria. Subjects had to be non-smokers, with a BMI between 20 and 30 kg/m^2^, not on medication, not premenopausal, following normal dietary habits (no specific diets, meals and food components.) and adhering to a wash-out period. Exclusion criteria included: not consuming more than four servings of fruits and vegetables per day, not having any type of food allergy or histamine intolerance, not displaying a high level of physical activity (defined as more than five training units/week), not having menstrual dysfunctions and not abusing alcohol.

All participants were informed about the purpose of the study via telephone calls or personal meetings. Subjects who wanted to join the study signed the informed consent form before their inclusion in the trial. Moreover, the selected subjects received a (poly)phenol-poor diet plan which they had to adhere to for 48 h before the first blood sampling. The list of permitted and forbidden foods is provided in Table S1. Relevant principles of Good Clinical Practice were followed throughout the study. The study was conducted according to the guidelines laid down in the Declaration of Helsinki, and all procedures involving human subjects were approved by the Ethics Committee of the Medical University of Graz, Austria, (EC-number: 27-507ex14/15). The trial was registered at www.clinicaltrials.gov (identifier No. NCT02587468).

### 2.2. Test Capsules

Capsules used for the study were provided by the Juice Plus Company/NSA LLC, Collierville, TN, USA and manufactured for Europe by Natural Alternatives International (NAI), Manno, Switzerland. The capsules contained powdered juice concentrate derived from 36 different fruits, vegetables, and berries including juice and pulp from different vegetal matrices, namely Juice PLUS+^®^ Vineyard (a berry blend), Juice PLUS+^®^ Fruit Blend and Juice PLUS+^®^ Vegetable Blend, which were kindly supplied by the Juice PLUS+^®^ company. In detail, the powder samples differed for their composition: Juice PLUS+^®^ Vineyard (hereafter called “berry blend”) contained 750 mg of dried powder blend of juice and pulp from grapes and berries (45.7%) including Concord grape, blueberry, cranberry, blackberry, bilberry, raspberry, redcurrant, blackcurrant, elderberry, in varying proportions, besides green tea, ginger root, grape seed, artichoke leaf powder, cocoa powder, and pomegranate powder. Juice PLUS+^®^ Fruit Blend (“fruit blend”) instead contained 750 mg of dried powder blend of juice and pulp (52%) of apple, orange, pineapple, cranberry, peach, acerola cherry, papaya, in varying proportions, beet root, date, and prune. Lastly, Juice PLUS+^®^ Vegetable Blend (“vegetable blend”) contained 750 mg of dried powder blend of juice and pulp (60%) of carrot, parsley, beet, kale, broccoli, cabbage, tomato, and spinach, in varying proportions, as well as sugar beet, garlic powder, oat, and rice bran. Moreover, the fruit and the vegetable powders were enriched with vitamins C, and folic acid) and with a natural carotenoid and tocopherol blend. The berry blend powder was enriched with vitamins C and folic acid as well as with a natural tocopherol blend.

### 2.3. Study Design

In an open one-arm study design, three capsules (one berry, fruit, and one vegetable blend), were consumed by each participant on one single occasion. Before the test meal, participants were asked to follow a two-week wash-out period avoiding all food supplements and dietetic products and all kinds of drugs/medications. Additionally, participants were asked to consume a (poly)phenol-poor diet 48 h before the test day. To facilitate participant adherence to the dietary restrictions, a list of permitted and forbidden foods was supplied. The day of the test, subjects visited the lab after an overnight fast, and after the baseline blood drawing (T_0_), they received the three capsules (one of each blend), to be consumed with 250 mL of still water. Four additional blood samples were collected, at 1, 2, 5, and 10 h after capsule intake. After the 2 h blood sampling, the subjects consumed a standardized (poly)phenol-poor snack, including white bread, cheese, ham, milk, and water *ad libitum*, in accordance to Pereira-Caro [30]. For each blood drawing, 5 mL of venous EDTA-blood from the elbow were collected via vein catheter, in supine position. Blood was immediately centrifuged at 2500 g for ten minutes to separate the plasma, which was frozen at −70 °C until uHPLC/MS^n^ analyses. 

### 2.4. Chemicals

All chemicals and solvents were of analytical grade. 3-Hydroxybenzoic acid, protocatechuic acid, 3-hydroxyphenylpropionic acid, hippuric acid, dihydrocaffeic acid, ferulic acid, dihydroferulic acid, (+)-catechin, 3-caffeoylquinic acid, 5-caffeoylquinic acid, quercetin 3-glucuronide, (hydroxyphenyl)-γ-valeric acid, and pyrogallol were purchased from Sigma-Aldrich (St. Louis, MO, USA). Dihydrocaffeic acid 3-*O*-sulfate, ferulic acid 4′-*O*-sulfate, dihydroferulic acid 4′-*O*-sulfate, caffeic acid 4′-*O*-glucuronide, dihydrocaffeic acid 3-*O*-glucuronide, isoferulic acid 3-*O*-glucuronide were purchased from Toronto Research Chemicals (Toronto, ON, Canada). (3′-hydroxyphenyl)-γ-valerolactone, (4′-hydroxyphenyl)-γ-valerolactone, (3′,5′-dihydroxyphenyl)-γ-valerolactone, (3′,4′-dihydroxyphenyl)-γ-valerolactone, (3′,4′,5′-trihydroxyphenyl)-γ-valerolactone, phenyl-γ-valerolactone-4′-*O*-sulfate, phenyl-γ-valerolactone-3′-*O*-sulfate, (5′-hydroxyphenyl)-γ-valerolactone-3′-*O*-sulfate, (4′-hydroxyphenyl)-γ-valerolactone-3′-*O*-sulfate, phenyl-γ-valerolactone-3′-*O*-glucuronide, phenyl-γ-valerolactone-3′,4′-di-*O*-sulfate, and (5′-hydroxyphenyl)-γ-valerolactone-3′-*O*-glucuronide were synthesized in house using the synthetic strategy previously outlined by Curti and colleagues [31] and following known procedures reported in the literature [32,33]. Feruloylglycine, 4-hydroxyhippuric acid, quercetin 3′-*O*-sulfate, hesperetin 3′-*O*-glucuronide, and hesperetin 7-*O*-glucuronide were kindly supplied by Professor Alan Crozier. Vanillic acid and (3-methoxy, 4-hydroxyphenyl)acetic acid were purchased from Alfa Aesar (Thermo Fisher (Kandel) GmbH, Postfach, Karlsruhe, Germany) and from Extrasynthese (Genay Cedex, France), respectively. Naringenin 4′-*O*-glucuronide and naringenin 7-*O*-glucuronide were purchased from Bertin Pharma (Montigny le Bretonneux, France). Ultrapure water from MilliQsystem (Millipore, Bedford, MA, USA) was used throughout the experiment.

### 2.5. Plasma Extraction

Plasma samples of all volunteers were extracted using a solid phase extraction (SPE) method as reported by Urpi-Sarda and colleagues, with some modifications [34]. Oasis^®^ HLB Vac cartridges (1 cc, 30 mg sorbent, 30 µm particle size) (Waters, Milford, Massachusetts, MA, USA) were conditioned with 1 mL of methanol and equilibrated with 1 mL of water. An aliquot of 800 μL of plasma was added with 16 μL of *o*-phosphoric acid (2.5%) and then loaded into cartridges. The cartridges were washed with 1 mL of acidified water (0.1% formic acid) and finally eluted with 1 mL of methanol containing formic acid (1.5 mol/L). The eluates were evaporated overnight to dryness by means of a rotary speed-vacuum at room temperature and reconstituted with 80 μL of methanol/acidified water (0.1% formic acid) (50:50 *v*/*v*) prior uHPLC/MS^n^ analysis.

### 2.6. UHPLC/MS^n^ Analysis

Plasma extracts were analyzed by a UHPLC DIONEX Ultimate 3000 equipped with a triple quadrupole TSQ Vantage (Thermo Fisher Scientific Inc., San Josè, CA, USA) fitted with a heated-ESI (H-ESI) (Thermo Fisher Scientific Inc., San Josè, CA, USA) probe. Separations were carried out by means of a Kinetex EVO C18 (100 × 2.1 mm) column, 1.7 μm particle size (Phenomenex, Torrance, CA, USA). 

For UHPLC, mobile phase A was acetonitrile containing 0.2% formic acid and mobile phase B was 0.2% formic acid in water. The gradient started with 5% A, isocratic conditions were maintained for 0.5 min, and reached 95% A after 6.5 min, followed by 1 min at 95%. The starting gradient was then immediately reestablished and maintained for 4 min to re-equilibrate the column. The flow rate was 0.4 mL/min, the injection volume was 5 μL, and the column temperature was set at 40 °C. 

The applied mass spectrometry (MS) method consisted in the selective determination of each target precursor ion by the acquisition of characteristic product ions in selective reaction monitoring (SRM) mode (Table 1), applying a negative ionization. 

To optimize the method, all the available standard compounds were infused into the MS to set the best mass parameters and to check the actual fragmentation patterns. Finally, for all the analyses, the spray voltage was set at 3 kV, the vaporizer temperature at 300 °C, and the capillary temperature operated at 270 °C. The sheath gas flow was 60 units, and auxiliary gas pressure was set to 10 units. Ultrahigh purity argon gas was used for collision-induced dissociation (CID). The S-lens values were defined for each compound based on infusion parameter optimization (Table 1). Conversely, for compounds that were not available for infusion, the S-lens values were set using the values obtained for the chemically closest available standards. Quantification was performed with calibration curves of standards, when available (Table 1). Data processing was performed using Xcalibur software (Thermo Scientific Inc., Waltham, MA, USA). All data were expressed as mean values ± SEM.

## 3. Results

### (Poly)Phenolic Compound Absorption

The capsules consumed in the present study were previously characterized for (poly)phenolic profile and quantified for their total phenolic content [23]. Capsules were made of 36 different plant matrices, and contained a wide array of different phenolic compounds, principally ellagitannins, flavan-3-ols, flavonols, and anthocyanins in berry blend capsules, flavones, flavonols, flavanones, and anthocyanins in fruit blend capsules, and flavones and flavonols in vegetable blend capsules.

Out of the 92 monitored molecules, 20 quantifiable metabolites were identified, or tentatively identified in plasma samples. All the quantified metabolites were found as conjugated compounds with sulfate, glucuronide or glycine moieties. Three glycine-conjugated metabolites, hippuric acid, 4-hydroxyhippuric acid, and feruloylglycine, were detected. A total of five metabolites were flavonol derivatives, including conjugates of quercetin, kaempferol, myricetin, and patuletin. Three metabolites were directly linked to flavanone metabolism, namely naringenin, and hesperetin derivatives, whereas only one flavone metabolite, namely diosmetin sulfate, was detected. Among flavan-3-ol derivatives, three conjugated phenyl‑γ-valerolactones were detected. Finally, five metabolites were small phenolic derivatives, including hydroxyphenylpropionic acid sulfate, ferulic acid glucuronide, pyrogallol sulfate, dihydroxybenzoic acid sulfate, and methyl-trihydroxybenzoic acid sulfate. Generally, with the exception of hippuric acid, plasma levels of the quantified metabolites did not exceed the nanomolar range. Considering those metabolites for which the origin is strictly attributable to (poly)phenolic compounds contained in the capsules, the most abundant metabolites resulted in the low molecular weight phenolics which are usually produced in the colon by microbial transformation of flavonoids.

The glycine-conjugated metabolites are ubiquitous and could originate both by endogenous precursors [18,35] and by microbial metabolism of phenolic compounds [36,37,38]. Their potential endogenous origin justifies the high concentration at baseline (Figure S1). 

Actually, the absorption curves of hippuric acid and 4-hydroxyhippuric acid did not show a notable concentration peak and their levels were basically constant during the study period. On the contrary, feruloylglycine exhibited a peak plasma concentration 2 h after consumption, indicating a stronger connection with the phenolic compounds introduced through the capsules, as only the capsules were consumed within the 2 h.

**Figure 1:**
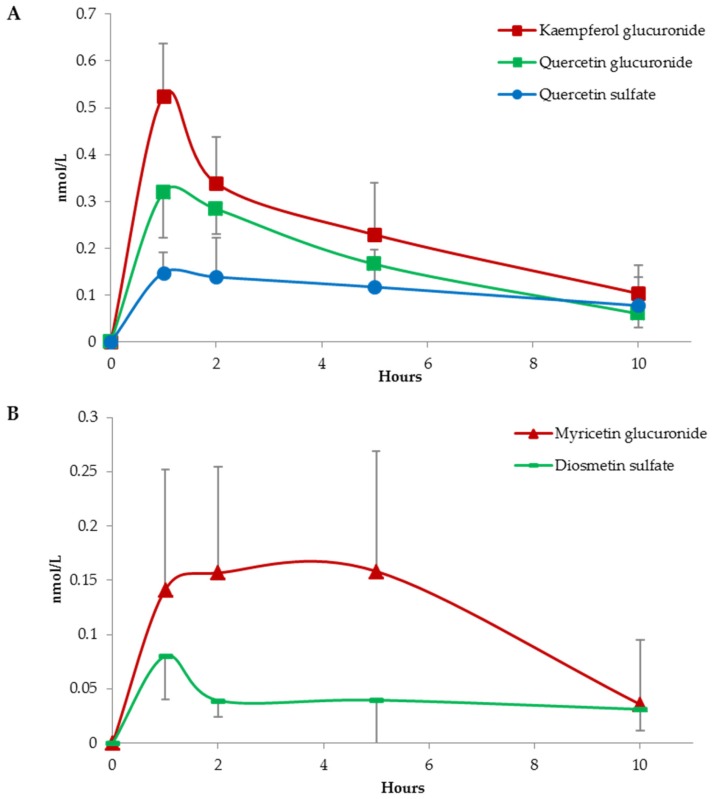
Absorption curves of kaempferol glucuronide, quercetin glucuronide, quercetin sulfate in graph (**A**); and myricetin glucuronide and diosmetin sulfate in graph (**B**). Data are expressed as mean values and bars represent standard error of means (SEM).

Looking to the other circulating metabolites appearing after capsule consumption, two metabolic phases could be easily distinguished. Conjugated metabolites appearing in the circulatory system within 1 or 2 h after capsule ingestion suggest an absorption in the first part of the gastro-intestinal tract. Native compounds are rapidly hydrolysed to release the aglycones, which are then conjugated by sulfotransferases (SULTs) and uridine-5′-diphosphate glucuronosyltransferases (UGTs) at both enterocyte and hepatic level before entering the systemic circulatory system [18]. Kaempferol glucuronide, quercetin glucuronide, quercetin sulfate, myricetin glucuronide, and diosmetin sulfate absorption curves are the expression of (poly)phenol metabolism in the first gastro-intestinal tract (Figure 1).

**Figure 2:**
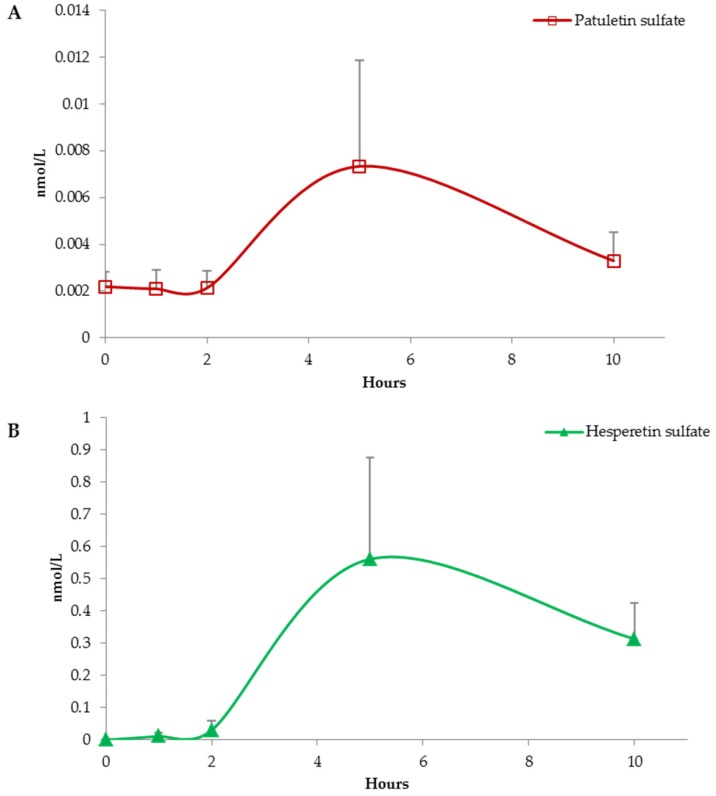
Absorption curves of patuletin sulfate in graph (**A**) and hesperetin sulfate in graph (**B**). Data are expressed as mean values and bars represent standard error of means (SEM).

A concentration peak recorded between 5 and 10 h after test meal indicates, instead, a clear interaction between the indigested (poly)phenolic fraction and the colonic microbiota. The absorption profile outlined by patuletin sulfate and hesperetin sulfate likely represents a colonocyte level absorption and a subsequent conjugation at hepatic level (Figure 2). Actually, their plasmatic concentration reached a maximum level 5 h post capsule consumption.

**Figure 3:**
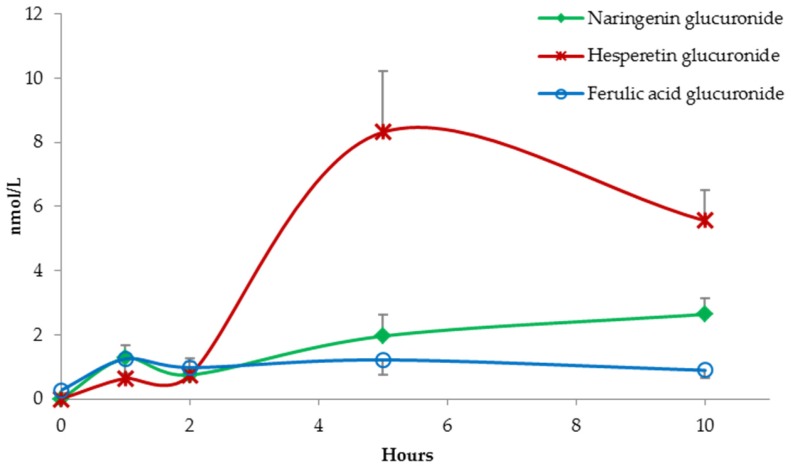
Absorption curves of naringenin glucuronide, hesperetin glucuronide, and ferulic acid glucuronide. Data are expressed as mean values and bars represent standard error of means (SEM).

Two quantified metabolites, namely naringenin glucuronide and hesperetin glucuronide, showed a double phase metabolism (Figure 3). 

Probably, naringenin and hesperetin were partially cleaved in the first gastro-intestinal tract and rapidly absorbed and metabolized at intestinal/hepatic level, resulting in a first peak approximately 1 h after capsule ingestion. However, the peak between 5 and 10 h indicates a more important role of the colonic microbiota in flavanone metabolism [30]. Similarly, ferulic acid glucuronide absorption profile showed a double phase curve (Figure 3). Nevertheless, no ferulic acid was detected in the capsules [23], suggesting that this compound probably originated by catechol-*O*-methyltransferase (COMT) activity on other hydroxycinnamic acids present in the capsules [39].

Finally, most (poly)phenolic compounds passing unmodified and unabsorbed through the first gastro-intestinal tract become a suitable substrate for the locally hosted microbiota. Several modifications on native compounds have been reported to be catalyzed by microbial enzymes, resulting in the formation of low molecular weight compounds [40], which are efficiently absorbed by colonocytes before hepatic conjugation. 

**Figure 4:**
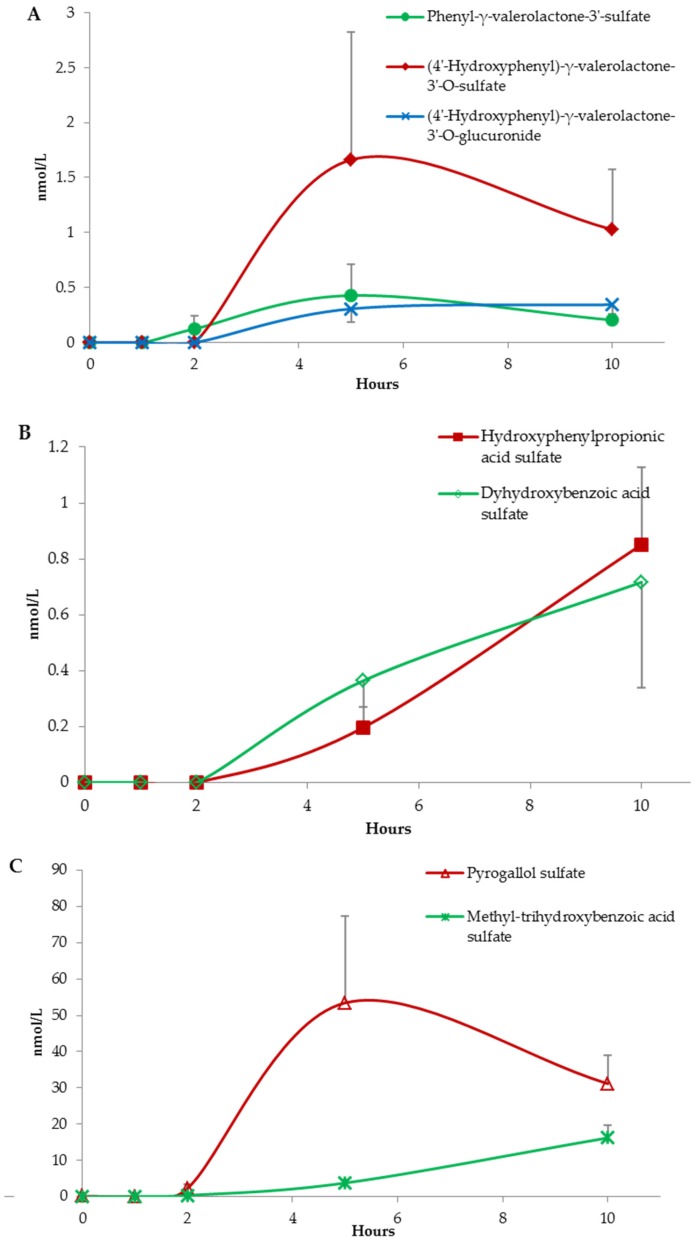
Absorption curves of phenyl-γ-valerolactone-3′-*O*-sulfate, (4′-hydroxyphenyl)-γ-valerolactone-3′-*O*-sulfate, and (4′-hydroxyphenyl)-γ-valerolactone-3′-*O*-glucuronide in graph (**A**); hydroxyphenylpropionic acid sulfate and (dihydroxybenzoic acid sulfate in graph (**B**); and pyrogallol sulfate and methyl-trihydroxybenzoic acid sulfate in graph (**C**). Data are expressed as mean values and bars represent standard error of means (SEM).

The three phenyl-γ-valerolactone derivatives, hydroxyphenylpropionic acid, pyrogallol, dihydroxybenzoic acid, and methyl-trihydroxybenzoic acid, having a peak concentration registered within 5 or 10 h, are typical metabolites generated by the host microbiota activity [41,42,43] (Figure 4).

## 4. Discussion

In the present study, the absorption profile of (poly)phenolic compounds derived from the ingestion of Juice PLUS+^®^ berry, fruit and vegetable blend capsules has been investigated. A total of 20 circulating metabolites have been identified, or tentatively identified, and quantified. As expected, detected metabolites derived from different (poly)phenols and appeared at different times in plasma. Flavonol metabolites principally originated in the first part of the gastro-intestinal tract, as previously reported by other authors. Feliciano and colleagues [41] reported a time to reach the maximum plasma concentration (T_max_) for kaempferol 3-*O*-glucuronide and quercetin 3-*O*-glucuronide of about 2 h. An absorption curve similar to those reported in the present study for quercetin 3-glucuronide and quercetin 3-sulfate was reported by Mullen and colleagues, who highlighted a concentration peak between 1 and 2 h after onion ingestion [44]. To the best of our knowledge, this is the first study in which plasma concentrations of patuletin and myricetin metabolites have been reported, suggesting that other flavonols could be effectively absorbed and hence investigated for their potential bioactivity. Moreover, a new flavone metabolite at plasmatic level has been detected, namely diosmetin sulfate. Its early peak plasma level registered at 1 h is in contrast with scientific data previously reported for other flavones, like apigenin. The T_max_ of apigenin (measured as aglycone after enzymatic hydrolysis) has been reported to be around 7 h after consumption of parsley [45]. The few human feeding studies involving flavones available so far reported a low bioavailability for this phytochemical [18,46], which could be ascribable to the low absorption of flavones, as demonstrated in the present investigation. Conversely, several scientific data are available for naringenin and hesperetin absorption, and all studies agree about the importance of colonic microbiota activity on this class of (poly)phenols [47,48,49]. The absorption curves observed in the present work confirm that flavanone metabolism principally occurs in the large intestine. Brett and colleagues reported a plasma concentration peak of flavanone conjugates 6 h after orange consumption [50], whereas the highest naringenin and hesperetin derivative urinary excretion has been reported within 2 and 10 h after orange juice consumption [30]. Flavan-3-ols were the most representative compounds in berry blend capsules [23]. However, no catechin monomer conjugates nor dimers or oligomeric proanthocyanidins were detected in plasma. However, in vivo studies have shown that both monomers and high molecular weight flavan-3-ols are effectively degraded by the gut microbiota into hydroxyphenyl-γ-valerolactones [51,52,53]. Many phenyl-γ-valerolactone derivatives have been detected after green tea [51,54], cocoa [34,55,56], wine [57] and almond [58] consumption. Partially compensating for the absence of flavan-3-ol monomers in plasma in the present study, three phenyl-γ-valerolactones were detected and quantified. 

It was then demonstrated that phenyl-γ-valerolactones represent an intermediate step in the microbial metabolism of flavan-3-ols and that other low molecular weight compounds, such as phenylacetic, phenylpropionic, benzoic acids derivatives [59] and hippuric acid, which derive from benzoic acids [60], could be formed. Likewise, flavonols, flavones, and flavanones, could be degraded into smaller phenolics, namely phenylacetic, phenylpropionic, and benzoic acid derivatives [61]. Similarly, anthocyanins undergo an important colonic set of transformations, giving rise to low molecular weight metabolites. After glucosidic cleavage of the sugar moiety, cyanidin could be the precursor of caffeic acid, from which ferulic and isoferulic acids could be formed after COMT activity [62,63,64]. Ferulic acid may undergo further phase II metabolism, namely sulfation and glucuronidation, generating conjugated ferulic derivatives [39,65]. Feliciano and colleagues recently hypothesized that peonidin could also lead to the formation of ferulic acids [41]. Moreover, anthocyanins could be converted into small phenolics such as phenylpropionic, phenylacetic, benzoic acids, and pyrogallol [62,64,66]. 

Concerning pyrogallol, the high plasmatic concentration of pyrogallol sulfate between 5 and 10 h recorded in the present study is in agreement with a T_max_ reported after cranberry consumption [41]. Gallic acid, which was present in the capsules, could have contributed to the formation of small metabolites [67], but also to other compounds like methylgallic acid [68], and, after phase II metabolism, to its 3-*O*-sulfate derivative [69]. 

**Figure 5:**
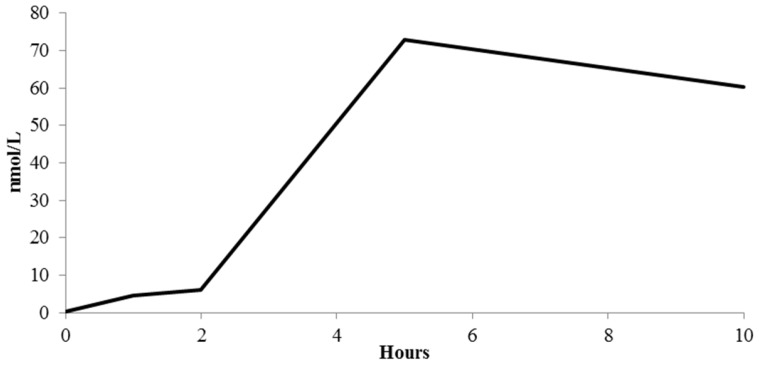
Total concentration of all circulating (poly)phenol metabolites, excluding hippuric acid, 4-hydroxyhippuric acid, and feruloylglycine. Data are expressed as mean values.

It now appears clear that the extensive bioconversion of (poly)phenol compounds strictly depends on the characteristics of individual colonic microbiota, and differences in microbiota composition now allow to discriminate phenotypes associated with producers and non-producers of specific metabolites [67,70,71]. As a matter of fact, inter-individual differences in the intestinal ecology may lead to differences in bioavailability, linked to specific metabolite production and, ideally, to differences in health benefits [72]. In the present study, the absorption of phenolics and the production of their metabolites is accompanied by a considerable inter-individual variability, plausibly due to the interaction between these compounds and the gut microbiota of the host. A clear example of this large variability among participants concerns the circulating concentration of phenyl-γ-valerolactones resulting from the catabolic transformations of catechins and procyanidins, operated mainly by *Clostridium coccoides* and *Bifidobacterium* spp. [73]. By analyzing the absorption curves of each participants, five subjects resulted as abundant producers of phenyl-γ-valerolactones, whereas the remaining subjects produced only extremely small amounts of these compounds (data not shown), suggesting marked variations in the colonic microflora of the individual volunteers. The inter-individual variability can affect not only the quantity of metabolites but also the timing of their appearance. For instance, the wide bars observed in the curves of kaempferol glucuronide are ascribable to the fact that these metabolites disappeared after 5 and 10 h in almost all the participants with the only exception of two subjects, who showed a second, later peak, probably due to colonic absorption. Considering this large variability, there is an increased interest in stratifying future study participants based on their polyphenol-metabolizing phenotypes (i.e., metabotypes) [72], and the present study supports the hypothesis that this variability should be carefully considered as a confounder of in vivo studies evaluating health effects of these phytochemicals. Finally, considering all the circulating detected metabolites, excluding those compounds whose origin could be attributed to endogenous precursors, such as hippuric acid, 4-hydroxyhippuric acid, and feruloylglycine [18,35], a “global” curve could be depicted to summarize the totality of quantified phenolic metabolites at every specific time point (Figure 5). Observing this graph, some considerations can be drawn: (i) the absorption and metabolism of (poly)phenols in the first gastro-intestinal tract (1–2 h after capsule ingestion) is low when compared to what occurs in the colon (5–10 h after capsule consumption); (ii) the plasma curves of the phenolic metabolites clearly highlighted the deep interaction between these compounds and the gut microbiota; (iii) the beneficial effects attributed to the regular consumption of these capsules does not depend on very high concentration of phenolic metabolites circulating after their consumption. In fact, plasmatic metabolites rarely exceeded nanomolar concentrations, as previously reported [18,19]. However, the effect of the regular and long time intake of these products may modify the way our organism interacts with the contained phenolics, perhaps improving its ability to absorb some of them at small intestinal level. Moreover, a modulation of the colonic microbiota in the long run seems plausible, perhaps in the direction of improved transformations, leading to increased absorption of microbial metabolites. Finally, the large variability observed in this short acute absorption study should be taken into consideration in future interventions, as not all the recruited volunteers might deal with Juice PLUS+^®^ phenolics in the same way. 

## 5. Conclusions

In conclusion, the consumption of Juice PLUS+^®^ Vineyard, Fruit, Vegetable blend, containing 36 different fruits, vegetables, and berries, allowed the effective absorption of several (poly)phenolic compounds. The capsules therefore ensure potential health-promoting molecules to be potentially available to target tissues and organs, presumably becoming responsible for the previously observed health effects. 

## Figures and Tables

**Table 1:** Spectrometric characteristics of the 92 monitored compounds, and standard compounds used for quantification of the 20 identified metabolites. Legend: SRM: selective reaction monitoring; ND: not detected.

Compound	[M − H]^−^	SRM Transition	S-Lens Value	Quantification
Catechol	109	108, 81	68	ND
Methylcatechol	123	108, 81	68	ND
Pyrogallol	125	124, 81, 97	68	ND
Hydroxybenzoic acid	137	91, 93, 45	70	ND
Hydroxyphenylacetic acid	151	107	51	ND
Dihydroxybenzoic acid	153	108, 109	64	ND
3-(3’-Hydroxyphenyl)propionic acid	165	119, 121	48	ND
Vanillic acid	167	152, 108, 123	60	ND
Gallic acid	169	125	68	ND
Hippuric acid	178	134	61	Hippuric acid
(3′-Methoxy, 4′-hydroxyphenyl)acetic acid	181	137	64	ND
Dihydrocaffeic acid	181	137, 119	64	ND
Methylgallic acid	183	168, 139	70	ND
Catechol sulfate	189	109, 81	70	ND
(3′-Hydroxyphenyl)-γ-valerolactone	191	147, 106	70	ND
(4′-Hydroxyphenyl)-γ-valerolactone	191	147, 173, 103, 107	70	ND
Ferulic acid	193	134, 178	71	ND
(Hydroxyphenyl)-γ-valeric acid	193	147, 149, 157, 175	72	ND
4-Hydroxyhippuric acid	194	100, 150	72	4-Hydroxyhippuric acid
Dihydroferulic acid	195	136	73	ND
Syringic acid	197	153, 182	70	ND
Methylcatechol sulfate	203	123, 108, 81	70	ND
Pyrogallol sulfate	205	125, 124, 81, 97	68	Dihydrocaffeic acid 3-*O*-sulfate
(3′,5′-Dihydroxyphenyl)-γ-valerolactone	207	163, 123, 121	75	ND
(3′,4′-Dihydroxyphenyl)-γ-valerolactone	207	163, 122	75	ND
(3′,5′-Dihydroxyphenyl)-γ-valeric acid	209	101, 124, 147	63	ND
(3′,4′-Dihydroxyphenyl)-γ-valeric acid	209	151, 165, 191, 194	63	ND
Hydroxybenzoic acid sulfate	217	137, 93, 45	70	ND
Methyl-trihydroxybenzoic acid sulfate	219	139, 124, 125, 81, 97	68	Dihydroferulic acid 4′-*O*-sulfate
(3′,4′,5′-Trihydroxyphenyl)-γ-valerolactone	223	179, 205, 138	75	ND
Dihydroxybenzoic acid sulfate	233	153, 108, 109	64	Dihydrocaffeic acid 3-*O*-sulfate
Hydroxyphenylpropionic acid sulfate	245	165, 121, 119	90	Dihydrocaffeic acid 3-*O*-sulfate
Vanillic acid sulfate	247	167, 152, 108, 123	90	ND
Gallic acid sulfate	249	169, 125	68	ND
Feruloylglycine	250	206, 134, 162, 191, 177	79	Feruloylglycine
Dihydrocaffeic acid sulfate	261	181, 137	96	ND
Methylgallic acid sulfate	263	183, 168, 125	68	ND
Phenyl-γ-valerolactone-4′-*O*-sulfate	271	191, 147	93	ND
Phenyl-γ-valerolactone-3′-*O*-sulfate	271	191, 147, 93, 80, 106	93	Phenyl-γ-valerolactone-3′-*O*-sulfate
Ferulic acid sulfate	273	193, 134, 178	92	ND
Phenyl-γ-valeric acid‑*O*‑sulfate	273	193, 175, 157, 149, 147	92	ND
Dihydroferulic acid sulfate	275	195, 136	75	ND
(5′-Hydroxyphenyl)-γ-valerolactone-3′-*O*-sulfate	287	207, 122, 163	96	ND
(4′-hydroxyphenyl)-γ-valerolactone-3′-*O*-sulfate	287	207, 109, 163	96	(4′-Hydroxyphenyl)-γ-valerolactone-3′-*O*-sulfate
(Epi)catechin	289	245, 203, 204.9	98	ND
(Hydroxyphenyl)-γ-valeric acid‑*O*‑sulfate	289	209, 191, 151, 147, 124, 101	92	ND
Dihydroxyphenyl-γ-valerolactone-*O*-sulfate	303	179, 223	90	ND
Methyl(epi)catechin	303	288, 245, 205	98	ND
Hydroxybenzoic acid glucuronide	313	137, 93, 45	70	ND
(Methyl-hydroxyphenyl)-γ-valerolactone-*O*-sulfate	317	222, 237	92	ND
Dihydroxybenzoic acid glucuronide	329	153, 108, 109	64	ND
Gallic acid glucuronide	345	169, 125	68	ND
Apigenin sulfate	349	269, 225	98	ND
Naringenin sulfate	351	271, 151	84	ND
3-Caffeoylquinic acid	353	191, 179, 135	85	ND
5-Caffeoylquinic acid	353	191	85	ND
Caffeic acid glucuronide	355	179, 135	87	ND
Dihydrocaffeic acid glucuronide	357	181, 137, 113	63	ND
Kaempferol sulfate	365	285, 257	90	ND
Phenyl-γ-valerolactone-3′-*O*-glucuronide	367	191, 113, 207	93	ND
Phenyl-γ-valerolactone-3′,4′-di-*O*-sulfate	367	287, 147	93	ND
Ferulic acid glucuronide	369	193, 178, 175	92	Isoferulic acid 3′-*O*-glucuronide
(Epi)catechin sulfate	369	289, 245, 203, 205	98	ND
Dihydroxyphenyl-γ-valeric acid disulfate	369	209, 191, 151, 147, 124	92	ND
Diosmetin sulfate	379	299, 284	90	Quercetin 3′-*O*-sulfate
Quercetin 3′-sulfate	381	301, 151, 179	83	Quercetin 3′-*O-*sulfate
Hesperetin sulfate	381	301, 151, 179	115	Quercetin 3′-*O*-sulfate
(4′-hydroxyphenyl)-γ-valerolactone-3′-*O*-glucuronide	383	207, 163	87	(5′-hydroxyphenyl)-γ-valerolactone-3′-*O*-glucuronide
Methyl(epi)catechin sulfate	383	303, 288, 245, 205	98	ND
(Epi)gallocatechin sulfate	385	305, 179, 221	98	ND
Myricetin sulfate	397	317, 316, 179	90	ND
Dihydroxyphenyl-γ-valerolactone‑*O*‑glucuronide	399	223, 175, 179	87	ND
Methyl(epi)gallocatechin sulfate	399	319, 304, 179, 221	98	ND
Patuletin sulfate	411	331, 316, 209	90	Quercetin 3′-*O*-sulfate
Spinacetin sulfate	425	345, 330	90	ND
Apigenin glucuronide	445	269, 225	90	ND
Naringenin 4′-glucuronide	447	271 151, 379, 119	112	Naringenin 4′-*O*-glucuronide
Naringenin 7-glucuronide	447	271, 151	84	ND
Kaempferol glucuronide	461	285, 257	90	Quercetin 3-*O*-glucuronide
Phenyl-γ-valerolactone‑3′,4′-*O*-sulfate‑*O*-glucuronide	463	163, 207, 287, 383	87	ND
(Epi)catechin glucuronide	465	289, 245, 205	98	ND
Diosmetin glucuronide	475	299, 284	90	ND
Quercetin 3-glucuronide	477	301, 151, 179	91	Quercetin 3-*O*-glucuronide
Hesperetin 3′-glucuronide	477	301, 113	115	ND
Hesperetin 7-glucuronide	477	301, 151	115	Hesperetin 7-*O*-glucuronide
Methyl(epi)catechin glucuronide	479	303, 288, 245, 205	98	ND
Hydroxyphenyl-γ-valerolactone‑*O*-sulfate‑*O*-glucuronide	479	303, 223, 175, 259	91	ND
(Epi)gallocatechin glucuronide	481	305, 179, 221	98	ND
Myricetin glucuronide	493	317, 316, 209	90	Quercetin 3-*O*-glucuronide
Methyl(epi)gallocatechin glucuronide	495	319, 304, 179, 221	98	ND
Patuletin glucuronide	507	331, 316, 209	90	ND
Spinacetin glucuronide	521	345, 330	90	ND

## Supplementary Materials

The following are available online at http://www.mdpi.com/2072-6643/9/3/194/s1, Table S1: List of forbidden and permitted foods during the (poly)phenol-poor diet. Figure S1: Absorption curves of (a) hippuric acid, (b) 4-hydroxyhippuric acid, (c) feruloylglycine. Data are expressed as mean values and bars represent standard error of means (SEM).
