# Supplementary material for: Absorption Profile of (Poly)Phenolic Compounds after Consumption of Three Food Supplements Containing 36 Different Fruits, Vegetables, and Berries
Source: Nutrients. 2017 Feb 26;9(3):194. doi: 10.3390/nu9030194 (PMC5372857; doi:10.3390/nu9030194)
Supplement: Supplementary file 1 [file nutrients-09-00194-s001.pdf]

Absorption Profile of (Poly)Phenolic Compounds after Consumption of Three Food Supplements Containing 36 Different Fruits, Vegetables, and Berries

Letizia Bresciani, Daniela Martini, Pedro Mena, Michele Tassotti, Luca Calani, Giacomo Brigati, Furio Brighenti, Sandra Holasek, Daniela-Eugenia Malliga, Manfred Lamprecht and Daniele Del Rio

Table S1. List of forbidden and permitted foods during the (poly)phenol-poor diet.

Forbidden Foods	Allowed Foods
Coffee and foods or beverages containing coffee	Milk, yogurt and ice cream (without chocolate, coffee, fruits, nuts and all ingredients reported in the forbidden list)
Cocoa and chocolate and sweet products containing cocoa or chocolate (such as biscuits, ice cream, pies, yogurt, beverages, snacks…)	Butter and margarine
Tea and foods or beverages containing tea	Refined cereal-based products (pasta, biscuits and pies without chocolate or coffee or fruits, refined bread and substitutes)
Infusions	Refined rice
Barley and beverages containing barley	Refined breakfast cereal (without chocolate, fruits or nuts)
Alcoholic drinks (such as wine and beer)	Meat and ham
Spirits aged in wood (such as whisky and rum)	Cheese
Fruits, dried fruit and fruit juices (such as oranges, apples, pears, berries, peaches, prunes, apricots, exotic fruits, cherries, grape…)	Fish
Nuts	Eggs
Seeds (sesame, poppy, sunflower,….)	Pizza without tomato
Vegetables and vegetable soup (all, particularly onion, cabbage, sauerkraut, broccoli, green pepper, carrots, except all the vegetables included in allowed foods)	Oil (if extra virgin olive oil: only one teaspoon per day)
Legumes (such as lentils, beans, peas, fava beans, soybean and soybean-based products, …)	Banana (max 1 per day) or melon (max 1 slice per day) or watermelon (max 1 slice per day) or pineapple (max 2 slices per day)
Potatoes	Salad (max 1 plate per day), eggplant (max 1 per day), red or yellow sweet pepper (max 1 per day), cucumber (max 1 per day), gherkin (max 5 per day), fresh mushrooms (max 100g per day), dried mushrooms (max 20g per day), and courgette (max 1 per day)
Olives	Tomato (allowed only the first day of the diet)
Wholemeal products (such as wholemeal pasta, cereal, bread and bread substitutes, biscuits, snacks)	Balsamic vinaigrette (one spoon per day)
Cereal and cereal products (rye, oat, corn, and others…. Refined wheat products are allowed! See below)	Honey (one spoon per day)

Spices and herbs (such as sage, rosemary, thyme, oregano, basil, mint, cloves, chives, dill, curry, cinnamon)	Soft drinks (excluding soft drinks with fruit, such as Fanta or lemonade)
Guarana and beverages containing guarana	
Tomato ketchup	
Jam, marmalade and jellies	
Muesli	
Maple syrup	
Peanut butter	
Apfelstrüdel and Sachertorte	

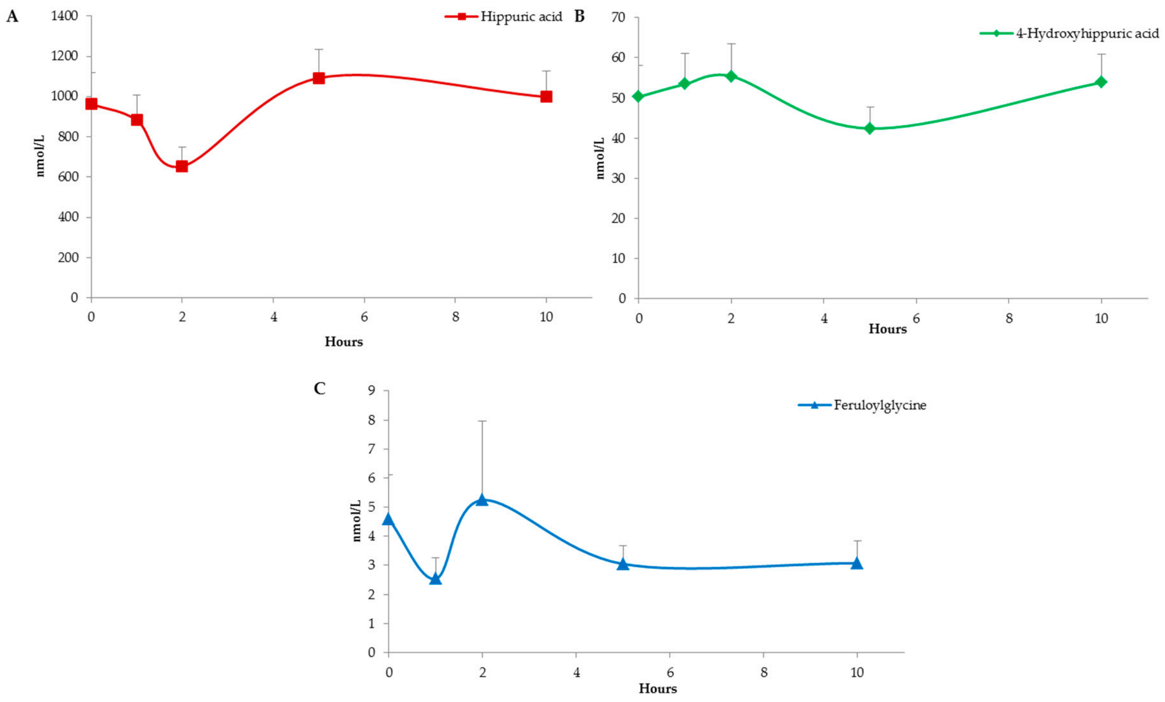

Figure S1. Absorption curves of (**A**) hippuric acid, (**B**) 4-hydroxyhippuric acid, (**C**) feruloylglycine. Data are expressed as mean values and bars represent standard error of means (SEM).
